# Biomarkers of Inflammation in Pediatric Arterial Ischemic Stroke

**DOI:** 10.15844/pedneurbriefs-30-11-1

**Published:** 2016-11

**Authors:** Mary Dunbar, Aleksandra Mineyko

**Affiliations:** 1 Department of Pediatrics, University of British Columbia, Vancouver, BC; 2 Department of Pediatrics and Neurosciences, University of Calgary, Calgary, AB

**Keywords:** Stroke, Inflammation, CRP, Varicella

## Abstract

Investigators from Switzerland studied inflammatory markers in children and neonates with acute arterial ischemic stroke (AIS).

Investigators from Switzerland studied inflammatory markers in children and neonates with acute arterial ischemic stroke (AIS). Twelve children and 6 term neonates with AIS were recruited through the Swiss Neuropediatric Stroke Registry program. Twenty-three inflammatory markers were analyzed. Results for children with AIS were compared to age-matched healthy controls. In childhood AIS, significantly higher concentrations of: MMP-9 (p = 0.04), TIMP-4 (p = 0.002), IL-6 (p = 0.003), IL-8 (p= 0.04), and CRP (p = 0.007) were seen than in controls. Stroke was associated with cerebral arteriopathy in 10 of 12 cases (83%). Five of the 12 children (42%) had VZV infection in the six months prior to AIS. These patients had significantly lower levels of IL-6 and CRP compared to the remaining seven without recent VZV infection, though still elevated compared to controls. There were significant differences in levels of MMP-1, MMP-2, TIMP-1, TIMP-2, TIMP-4 sE-selectin, sICAM-1, sVCAM-1, IL-8, IL-10, TNFα, VEGF, Fetuin and haptoglobin measured in neonates was compared to older children with AIS. The authors propose there is a pro-inflammatory state with specific biomarker elevations associated with acute arterial ischemic stroke in both pediatric and neonatal populations. It is unclear if inflammation is an aspect of the pathophysiology of stroke, or a consequence of tissue injury. [1]

COMMENTARY. Biomarkers of cerebral injury in stroke are of great prognostic interest, but none have been identified with clear associations with stroke outcome. Markers of inflammation have been of particular interest due to their proposed role in ongoing parenchymal injury and the increased stroke risk in children with recent infection. The recent, multicenter VIPS study (Vascular effects of Infection in Pediatric Stroke) [2] enrolled 355 patients ages 29 days to 19 years with AIS and evaluated high sensitivity CRP (hsCRP) and serum amyloid (SAA) within one month of stroke symptoms. It was found that higher concentrations of hsCRP and SAA were associated with an increased hazard ratio of recurrent AIS in children with arteriopathy.

Varicella infection within the previous year of stroke has been associated with transient arteriopathy. Initial studies demonstrated similar long-term outcome compared to non-varicella associated stroke, but with a significantly increased risk of early stroke recurrence [3].

Risk factors for neonatal AIS favor inflammatory etiologies, including chorioamnionitis, prolonged rupture of membranes, and other systemic stressors such as hypoglycemia and low APGARs [4]. Recurrence of stroke is rare, reflecting the transient nature of these conditions.

The current study adds to this knowledge by demonstrating a clear and statistically significant pattern of elevation of numerous biomarkers in children with predominantly arteriopathic AIS compared to age-matched controls. In addition, there appears to be a distinct pattern associated with recent varicella infection. Finally, the study also highlights the differences in neonatal stroke biomarkers compared to an older pediatric population. These results support ongoing research regarding the role of inflammation in stroke pathogenesis and of inflammatory biomarkers in prognostication and treatment.
